# Coloration and Fire Retardancy of Transparent Wood Composites by Metal Ions

**DOI:** 10.1021/acsami.3c13585

**Published:** 2023-12-06

**Authors:** Pratick Samanta, Archana Samanta, Lorenza Maddalena, Federico Carosio, Ying Gao, Céline Montanari, Mathias Nero, Tom Willhammar, Lars A. Berglund, Yuanyuan Li

**Affiliations:** † Department of Fibre and Polymer Technology, Wallenberg Wood Science Center, KTH Royal Institute of Technology, Stockholm 100 44, Sweden; ‡ Department of Applied Physics, KTH Royal Institute of Technology, Stockholm 114 19, Sweden; § Dipartimento di Scienza Applicata e Tecnologia, Politecnico di Torino, Alessandria Campus, Viale Teresa Michel 5, 15121 Alessandria, Italy; ∥ Jiangsu Co-Innovation Center of Efficient Processing and Utilization of Forest Resources, Nanjing Forestry University, Nanjing 210037, China; ⊥ Department of Materials and Environmental Chemistry, Stockholm University, SE-106 91 Stockholm, Sweden

**Keywords:** metal ion, methyl methacrylate (MMA), colored transparent wood, fire retardancy, scale-up

## Abstract

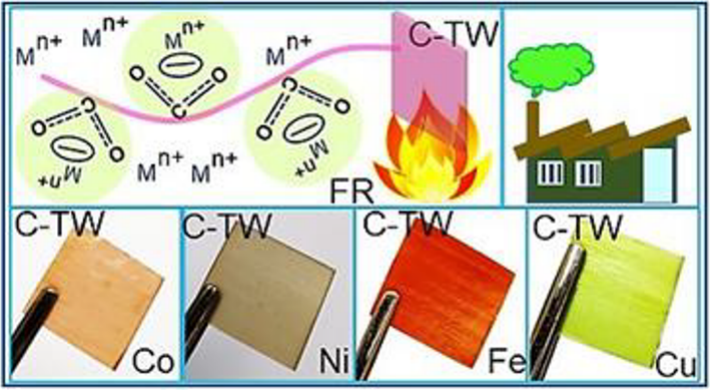

Transparent wood composites (TWs) offer the possibility of unique coloration effects. A colored transparent wood composite (C-TW) with enhanced fire retardancy was impregnated by metal ion solutions, followed by methyl methacrylate (MMA) impregnation and polymerization. Bleached birch wood with a preserved hierarchical structure acted as a host for metal ions. Cobalt, nickel, copper, and iron metal salts were used. The location and distribution of metal ions in C-TW as well as the mechanical performance, optical properties, and fire retardancy were investigated. The C-TW coloration is tunable by controlling the metal ion species and concentration. The metal ions reduced heat release rates and limited the production of smoke during forced combustion tests. The potential for scaled-up production was verified by fabricating samples with a dimension of 180 × 100 × 1 (*l* × *b* × *h*) mm^3^.

## Introduction

Transparent wood composites (TWs) have received significant attention because of high mechanical properties, light weight, and attractive optical properties.^1,2^ The low thermal conductivity further enhances its potential in energy-efficient buildings. A transparent wood composite (TW) is normally prepared by a first step of delignification^1^ or bleaching^3^ to remove lignin chromophores in native wood (NW) and minimize light absorption. Then, impregnation and polymerization of a monomer are carried out where the polymer refractive index (RI) is matched to the wood substrate. The TW was first proposed for wood anatomy studies in 1992.^4^ Technical relevance was then suggested.^1,2^ The state of the art was described in 2018,^5^ and the topic is also covered in a more recent general wood materials review.^6^ Today, there is a strong interest in functional transparent biocomposites with structural performance for applications in thermal energy storage,^7^ electronic devices,^8^ EMI shielding,^9^ heat shielding,^10^ fire retardancy,^11^ and photonic^12,13^ devices. In addition, investigation of fire retardancy that is highly related to practical application is often missed for C-TWs, although there are plenty of studies focused on fire-retardant TWs.^14^

Colored transparent wood is attractive for load-bearing aesthetic design applications such as colorful windows, lighting devices, design furniture, etc. Direct monomer infiltration and polymerization in native wood would also result in C-TW, although in native wood color from lignin and extractives.^1,3^ White delignified/bleached wood provides more design freedom since pigments,^15^ dyes,^16^ and inorganic nanoparticles with a structural color^13,17^ can be added. For example, red C-TW was prepared by incorporation of the Rhodamine 6G (Rh6G) organic dye molecules in the wood structure.^18^ Thermochromic^19^ and photochromic^20^ dyes were also used for functional colored transparent wood composites (C-TWs). However, the durability of conventional dyes and pigments against photobleaching may be a concern. In this respect, structural color from plasmonic nanoparticles provides brilliant color and photostability.^21−24^ This requires uniform size and distribution of the nanoparticles in wood, which is a challenge.^25,26^ In situ nanoparticle synthesis is an option, although processing becomes more complex.

Transition metal salts are interesting alternatives and give a bright color after coordination with ligands such as water.^27−29^ The color could be tuned by changing the concentration or the metal ion species. Wood contains functional groups like hydroxyl, phenolic, and carboxyl, which may bind metal ions, although the mechanism is not fully understood.^30−34^ So far, the most putative mechanisms suggested are ion exchange, coordination, complexation, adsorption, etc.^35^ When a bleached wood (BW) substrate is immersed in a colored water-metal ion solution, the wood is transformed into brightly colored wood (CW). These CW substrates could be considered directly for the preparation of C-TWs. Here, C-TW was obtained through metal ion introduction, followed by methyl methacrylate (MMA) impregnation and polymerization. The composite microstructure is characterized for the purpose of better understanding of processing–structure and structure–property relationships. Additional improved fire retardancy was found due to the presence of metal ions. The possibility of scale-up of this method is investigated through the development of a large C-TW sample with a dimension of 180 × 100 × 1 (*l* × *b* × *h*) mm^3^.

## Experimental Section

### Materials

Birch wood with an oven dry weight density of 577 kg/m^3^ from Sydfanér AB, Sweden, was used in this study. Sodium acetate, hydrogen peroxide (H_2_O_2_) (30%), sodium hydroxide, sodium silicate, magnesium sulfate, 2,2′-azobis (2-methylpropionitrile) (AIBN), methyl methacrylate (MMA), and metal salts (cobalt(II) nitrate hexahydrate, copper(II) chloride dihydrate, nickel(II) nitrate hexahydrate, and iron(III) chloride hexahydrate) were purchased from Sigma-Aldrich. Acetone (99.5%) and ethanol absolute (99.8%) were bought from VWR, Sweden. Dimethylallyltriamine pentaacetic acid (DTPA) was received from Acros Organics and used as received.

### Chemical Modifications of Wood

Birch wood templates with a dimension of 20 × 20 × 1 (*l* × *b* × *h*) mm^3^ were chemically modified using bleaching chemicals including sodium silicate (3.0 wt %), hydrogen peroxide (4.0 wt %), sodium hydroxide (3.0 wt %), magnesium sulfate (0.1 wt %), and DTPA (0.1 wt %) in deionized water (DI) at 70 °C until the sample became bright white according to our previous report.^3^

### Preparation of Colored Transparent Wood Composites (C-TWs)

**Figure 1:**
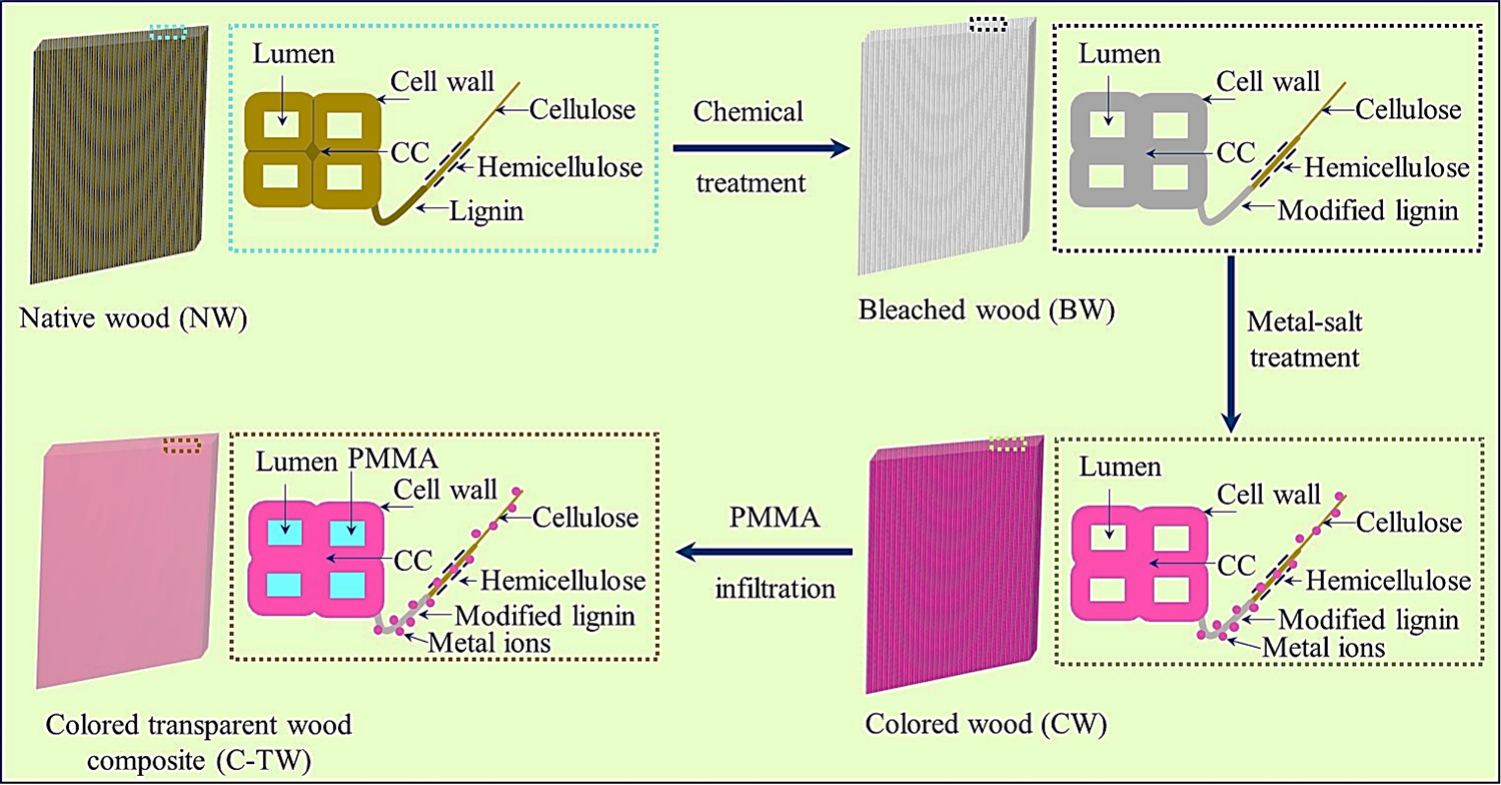
Schematic representation of the preparation of colored transparent wood using metal salts.

The chemically modified birch bleached wood (BW) was washed with DI water thoroughly to remove traces of bleaching chemicals and then washed with ethanol followed by acetone under vacuum condition to dehydrate the wood template. Subsequently, the acetone-immersed BW was transferred to the prepared colored metal salt solution for 2 h under vacuum condition. Metal salt solutions were prepared by adding metal salts in DI water and then stirring for 15 min with a magnetic stirrer for complete dissolution. The concentrations and salt species are listed in Table 1. Through the infiltration, the metal ions diffused and combined with BW to develop colored wood (CW). The CW was dehydrated again through the solvent exchange with ethanol, then followed by acetone under vacuum condition. Later, the colored wood was transferred from acetone to partially polymerized MMA (prepolymer) solution containing (0.5 wt %) AIBN under vacuum for 24 h. The impregnated CW was sandwiched between two glass slides and polymerized slowly using step heating from 35 and 45 °C to 75 °C for 6, 6, and 12 h, respectively. The schematic of preparation C-TW using metal salt is shown in Figure 1. The weight of the BW was calculated before and after metal salt treatment. Based on weight difference, metal ions (wt %) contained in CW were measured. After infiltration and MMA polymerization inside CW, the weight of the prepared composite (C-TW) was measured again to calculate the PMMA loading. According to the weight of metal ions, PMMA, and BW, the wood contained (wt %) in C-TW was calculated. The cobalt(II) nitrate hexahydrate metal salt was taken as the model system here. In addition, with DI water, this salt gives a pinkish red-colored solution (Figure S1). The cobalt(II) nitrate hexahydrate metal salt concentration was increased from 0.1 to 1 M in the solution to increase the depth of color of the C-TW.

**Table 1:** Codes of Transparent Wood Composites without and with Metal Ions Are Decided Based on Their Processing Method

wood	modification	metal salt	conc. in water (M)	polymer	code
birch	bleaching			PMMA	TW
		cobalt(II) nitrate hexahydrate	0.1		TW0.1Co
			0.5		TW0.5Co
			1.0		TW1.0Co
		nickel(II) nitrate hexahydrate	0.1		TW0.1Ni
		copper(II) chloride dihydrate	0.1		TW0.1Cu
		iron(III) chloride hexahydrate	0.1		TW0.1Fe

To display the possibility of the preparation of C-TWs from other metal salts, copper(II) chloride dihydrate, nickel(II) nitrate hexahydrate, and iron(III) chloride hexahydrate metal salts with a concentration of 0.1 M were used. Once, the copper(II) chloride dihydrate, iron(III) chloride hexahydrate, and nickel(II) nitrate hexahydrate metal salts were added individually in DI water, and solutions looked light blue-, dark yellow-, and light green-colored, respectively (Figure S1). The colored woods were prepared after dipping the BW into these colored metal salt solutions under vacuum condition for 2 h. Later, loosely adhered metal ions and salt from the surface of colored woods (CWs) were cleaned with DI water washing. Further, the CWs were dehydrated again by ethanol wash, followed by acetone rinse. MMA was infiltrated and polymerized in these colored woods in a similar way as mentioned in the above section to prepare C-TWs. The samples codes are mentioned in Table 1.

## Characterization

### Scanning Electron Microscopy (SEM) and Energy Dispersive X-ray (EDX) Analysis

The samples were freeze-fractured in liquid nitrogen. Cross-sectional images were obtained by field-emission scanning electron microscopy (FE-SEM, Hitachi S-4800, Japan). The surface of the samples was coated with 1 nm thick platinum–palladium for SEM and EDX analysis. The cross section was observed at an operating accelerating voltage of 1 kV. The EDX mapping of elements on freeze-fractured surfaces was carried out with an EDX detector (X-Max^N^, Oxford Instruments). The EDX mapping was conducted at an accelerating voltage of 15 kV.

### Scanning Transmission Electron Microscopy (STEM) with Energy Dispersive X-ray (EDX) Analysis

The scanning transmission electron microscopy (STEM) micrographs were acquired by a double Thermo Fisher Themis Z transmission electron microscope. The microscope was operated with an accelerating voltage of 300 kV. To study the internal morphology of the TW and C-TWs, thin cross sections were prepared by the Leica Ultracut UCT with a diamond knife (45°) from Diatome with a clearance angle of 6°. The samples were sectioned at a speed of 1 mm s^–1^. The estimated thickness of each section was ca. 100 nm. The thin sections were transferred to carbon-coated copper grids (EMS CF 400-CU-UL) for STEM with EDX analysis.

### Fourier-Transform Infrared Spectroscopy (FTIR)

The BW, CWs, TW, and C-TWs were characterized by the FTIR instrument (spectrum 100 Fourier transform infrared (FTIR) from PerkinElmer) with an additional attachment of an ATR diamond crystal (Graseby Specac Ltd. UK) and an MKII Golden Gate. The spectra were recorded from 4000 to 600 cm^–1^ at room temperature.

### Crystallinity Index (CI)

The crystallinity index was measured by a powder X-ray diffractometer (PANalytical, X’Pert PRO). Samples were scanned at (2θ) 5–80° with a step of size of 0.04°. The sample analysis was performed with Cu Kα radiation at 40 mA and 45 kV.

### Mechanical Properties

The tensile strength and elastic modulus of NW, BW, TW, and C-TWs were measured using an Instron 5944 (USA) instrument with a load cell of 2 kN. The gauge length was 25 mm. During the tensile test, a strain rate of 10%/min was maintained. All the samples were conditioned at 22 ± 1 °C with 50 ± 2% relative humidity (R.H.) for 24 h before the measurement. The sample with a dimension of 50 × 5 × 1 (*l* × *b* × *h*) mm^3^ was used for the test.

### Optical Properties

The optical performance including transmittance and haze was measured based on the ASTM D1003-00 standard test method^36^ with a wide range of wavelength light source coupled with an integrating sphere. The excitation source, a laser-driven xenon plasma white light source (Energetiq EQ-99), was coupled with an adjustable monochromator (SP2150i, Princeton Instruments). The integrating sphere (Lab sphere) with a diameter of around 15 cm was used for light collection. For the signal accumulation, a Peltier element-cooled CCD camera (−75 °C, Princeton Instruments) was linked with a spectrophotometer. The multimode optical fibers were used to attach the setup with an integrating sphere. The sample was placed on an opening port diameter of 13 mm during the measurement. The surrounding noise was subtracted during the measurements.

### Fire-Retardancy Test

Cone calorimetry (Noselab, ats-fire testing) was employed to investigate the combustion behavior of 50 × 50 × 1 (*l* × *b* × *h*) mm^3^ square samples under 35 kW/m^2^ radiative heat flux. The measurements were performed three times for each formulation for evaluating the “time to ignition” (TTI, s), average and peak of heat release rate (avHRR and pkHRR in kW/m^2^), total heat release (THR, MJ/m^2^), effective heat of combustion (EHC, MJ/kg), smoke production rate (SPR, m^2^/s), total smoke release (TSR, m^2^/m^2^), and final residue. Prior to cone calorimetry tests, all specimens were conditioned at 23 ± 1 °C for 48 h at 50% R.H. Cone calorimetry residues were characterized by means of SEM (SEM, Zeiss Evo 15, Jena, Germany) equipped with EDS (Oxford Ultimax 40) and XRD (PANalytical X’Pert MPD PRO) diffractometers (PANalytical, Almelo, Netherlands), Bragg–Brentano geometry, and Cu Kα radiation (λ = 1.5419 Å; scan range, 30–50°; scan rate, 4°/min).

## Results and Discussion

### Morphology and Chemical Analysis

**Figure 2:**
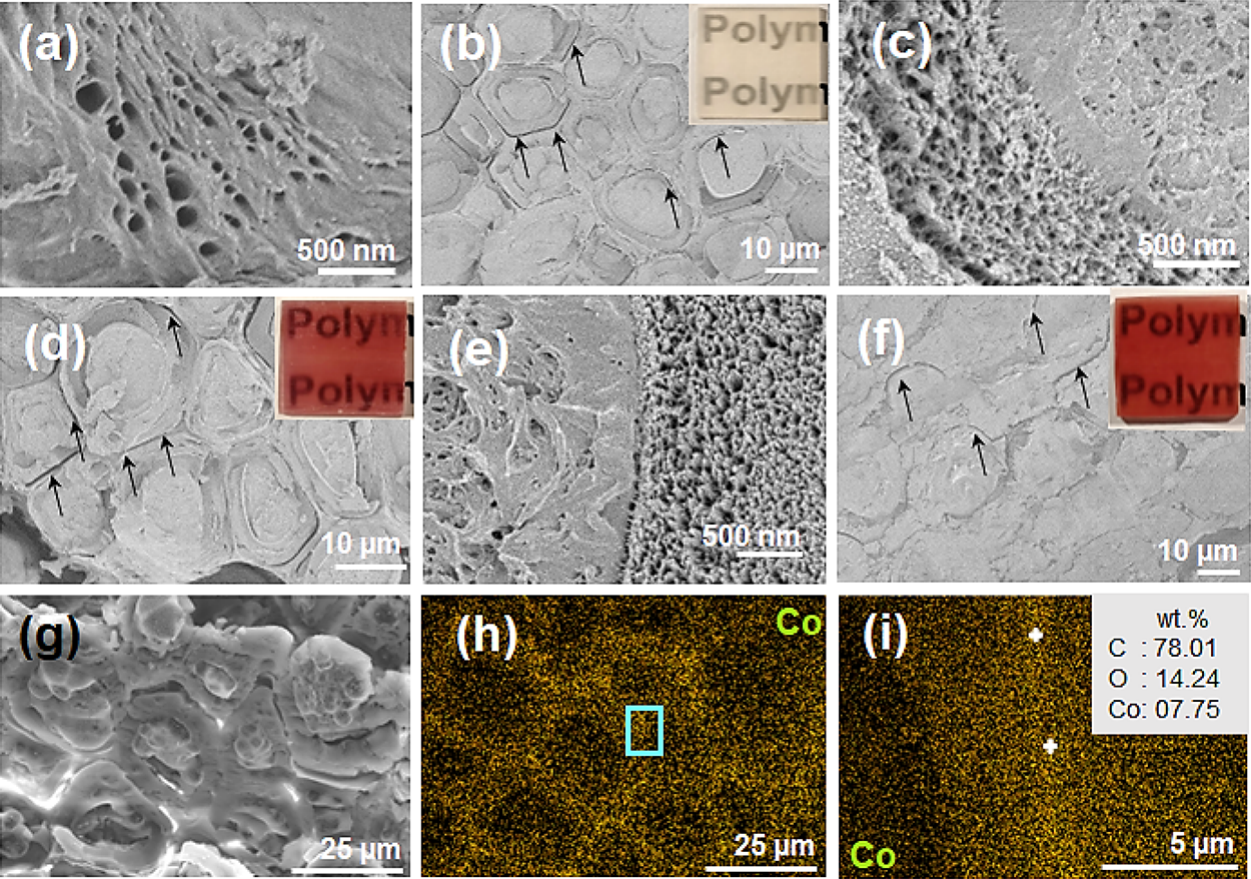
Cross-sectional micrographs of bleached wood (BW) and colored transparent wood composites (C-TWs). (a) Enlarged view of the BW cell wall. (b) TW is placed on printed paper (inset). (c) Enlarged view of the TW cell wall. (d) TW0.1Co is placed on printed paper (inset). (e) Enlarged view of the TW0.1Co cell wall. (f) TW1.0Co is placed on printed paper (inset). EDX analysis of TW0.1Co: (g) surface morphology, (h) relative mapping with the Co element, and (i) enlarged view of relative mapping with the Co element near middle lamella. In panels (b), (d), and (f), the black arrows suggest wood-polymer debond cracks.

Figure S2 shows the morphology of native birch with fibers, vessels, and ray cells in hollow tubular shaped structures as well as the cell wall structure. The cell walls contain brownish lignin (∼20 wt %^37^), which is responsible for 80–95% of absorbed^1^ light. The lignin phase in the NW was chemically modified (bleached) using green H_2_O_2_ to decrease light absorption. Bleached wood (BW) shows a well-preserved macrostructure (Figure S2). During the process, chromophores in lignin are selectively removed, leading to bright white wood samples (inset, Figure S2). Because of the bleaching approach, lignin-rich regions such as cell wall corners were better preserved as compared to delignified samples reported in the literature.^1,3,37,38^ The enlarged cell wall region image shows nano- and mesoscale porosity due to the partial loss of lignin and hemicelluloses (Figure 2a).

The bleached birch wood and colored wood (bleached wood impregnated by ions) are optically opaque (Figure S3) due to the strong scattering of light from micro-, meso-, and nanoscale pores in the wood structure.^1^ The modified wood substrates were then impregnated by a liquid MMA monomer for polymerization to provide high optical transmittance. The cross-sectional view of the resulting TW is shown in Figure 2b, and a TW sample on printed paper is shown in Figure 2b (inset). The cross-sectional micrographs show that all lumen space (central pore space in fibrous cells) was completely filled with PMMA. Microdefects in the form of debond gaps (marked with black arrows in Figure 2b) were noticed. A higher magnification view (Figure 2c) illustrates the nanoporous nature of the cell wall in this TW specimen. The cross-sectional view of a colored transparent wood composite (TW0.1Co) prepared from 0.1 M cobalt salt solution is shown in Figure 2d, and this TW0.1Co sample is shown on printed paper in Figure 2d (inset). TW0.1Co showed a bright red color because of cobalt ions. The lumen space of TW0.1Co was completely filled by PMMA, and cell wall porosity (Figure 2d,e) appeared similar to that of TW (Figure 2d). Colored transparent wood from higher concentration cobalt salt solutions such as 0.5 M (TW0.5Co) and 1.0 M (TW1.0Co) also showed complete filling of the lumen space by PMMA and showed nanoscale cell wall porosity (Figure S4 and Figure 2f). The TW0.5Co and TW1.0Co samples placed on printed paper are shown in Figure S4 and Figure 2f (inset), respectively. The saturation of red color in C-TWs increased with increasing cobalt salt concentration from 0.1 to 1.0 M, which means that color saturation can be controlled.

The cobalt ion location in the C-TWs was studied by EDX mapping. The cross sections of TW and TW0.1Co were mapped by Co elements, and the mapped images are shown in Figure S5 and Figure 2g,h, respectively. No Co signal was detected in the TW control. For TW0.1Co, the Co signal was located in the cell wall region. A magnified image shows a stronger Co signal in the middle lamella region between fiber cells (Figure 2i). This suggests the preferential migration of cobalt ions to lignin-rich regions. The point scan method was used to calculate Co weight fraction in the middle lamella of TW0.1Co (Figure 2i, marked with white-colored “+”). The relative weight percentage of Co was ∼7.75 for TW0.1Co. Similar information was obtained from TW0.5Co and TW1.0 Co samples (Figure S6).

**Figure 3:**
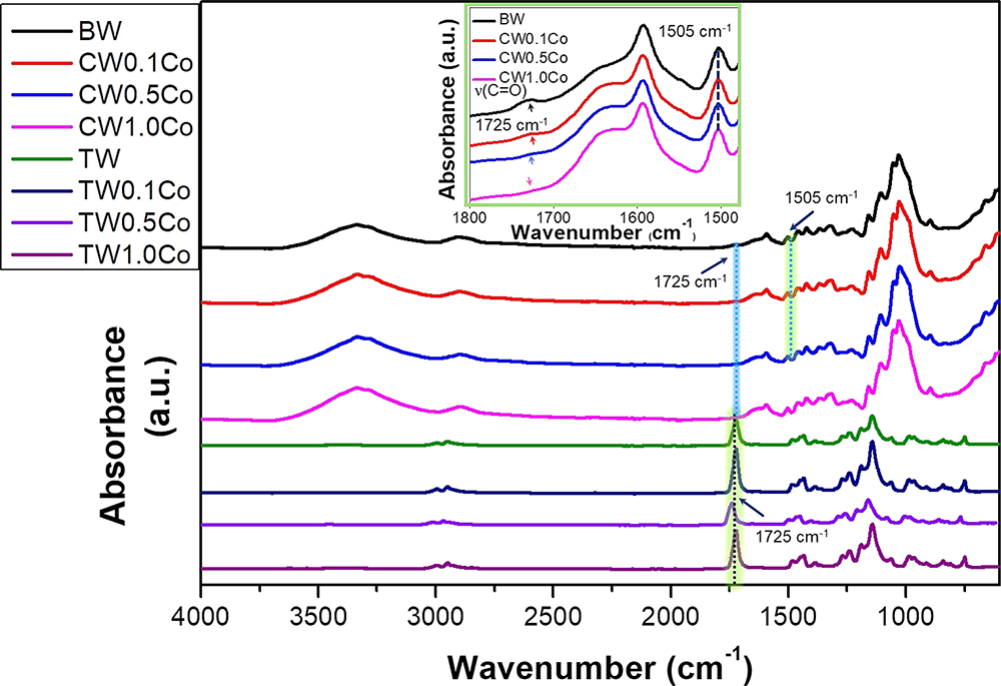
FTIR spectra of chemically modified bleached wood (BW), colored woods (CWs) with different metal ions, and TW and colored transparent wood composites (C-TWs). The inset shows the enlarged view of FTIR spectra of BW and CWs.

**Figure 4:**
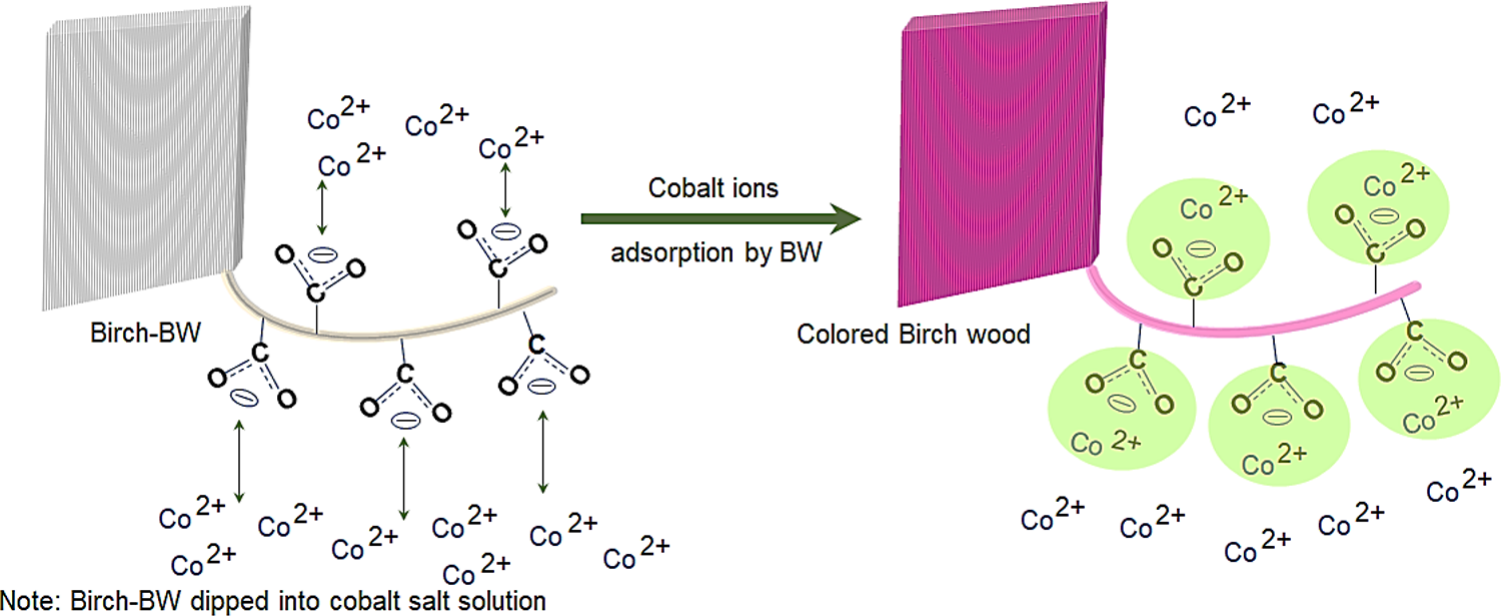
Schematic illustration of the mechanism of the combination of metal ions with BW wood for preparation of CW.

The bleached wood template was dipped in a cobalt nitrate hexahydrate salt solution to prepare colored wood. During this process, the solution pH was about 6–7. In this pH range, functional groups like carboxyl groups of xylan in hemicellulose and carboxyl groups of pectin in primary wall and lignin may bind to metal ions.^35,39^ The interaction of cobalt ions with wood was investigated through FTIR analysis. The FTIR spectra of BW and CWs infiltrated with different cobalt nitrate hexahydrate salt solutions are shown in Figure 3. The stretching at 1505 cm^–1^ represents the presence of aromatic rings in lignin.^7^ This peak was noticed for all samples (inset of Figure 3). The stretching of C=O bonds in carboxyl groups is located at 1725 cm^–1^ (inset of Figure 3). This peak intensity decreased, and the peak position shifted marginally to 2–3 cm^–1^ after infiltration with 0.1 M cobalt salt solution. It suggests the possibility of ionic interactions between cobalt ions and carboxyls in bleached wood.^35^ Carboxyl peak intensity decreased with increased cobalt salt concentration from 0.1 to 1 M (inset of Figure 3). The proposed mechanism of ionic interactions between cobalt ions and carboxyls in BW is shown in the schematic sketch in Figure 4. When the BW is immersed in cobalt salt solution, the carboxyl groups have an electronic cloud between two C–O bonds, hence in between one negative charge.^35^ Because of electronic affinity, COO^–^ groups attract Co^2+^ metal ions and then combine through ion exchange to form CW. The cobalt content in CW was measured via weight change. The cobalt ion content in CW0.1Co was ca. ∼1.6 wt % (Figure S7), and the cobalt ion content increased in CW with increased cobalt salt concentration (ca. ∼3.4 and ca. ∼3.9 wt % in CW0.5Co and CW1.0Co, respectively). The FTIR spectra of C-TW (TW0.1Co, TW0.5Co, and TW1.0Co) showed an additional strong adsorption peak at 1725 cm^–1^ (Figure 3). This is due to the stretching of C=O groups in PMMA.^7^

Wide-angle X-ray diffraction was used to check for any presence of cobalt nanoparticles in the C-TWs. The WXRD patterns of TW, TW0.1Co, TW0.5Co, and TW1.0Co samples are shown in Figure S8. The cellulose I crystal structure shows diffraction peaks at 14.7° and 22.6°.^40^ All of the samples showed these characteristic cellulose peaks, which confirms that the cellulose crystal structure is unaffected by the salt treatment. Cobalt ions are probably associated with the amorphous hemicellulose and lignin structures. The intensity of the peaks is somewhat broadened since the wood pore space is filled by amorphous PMMA. Since there were no additional WAXD peaks and no nanoparticles visible in FE-SEM cross sections, cobalt ions are not likely to have formed nanoparticles.

**Figure 5:**
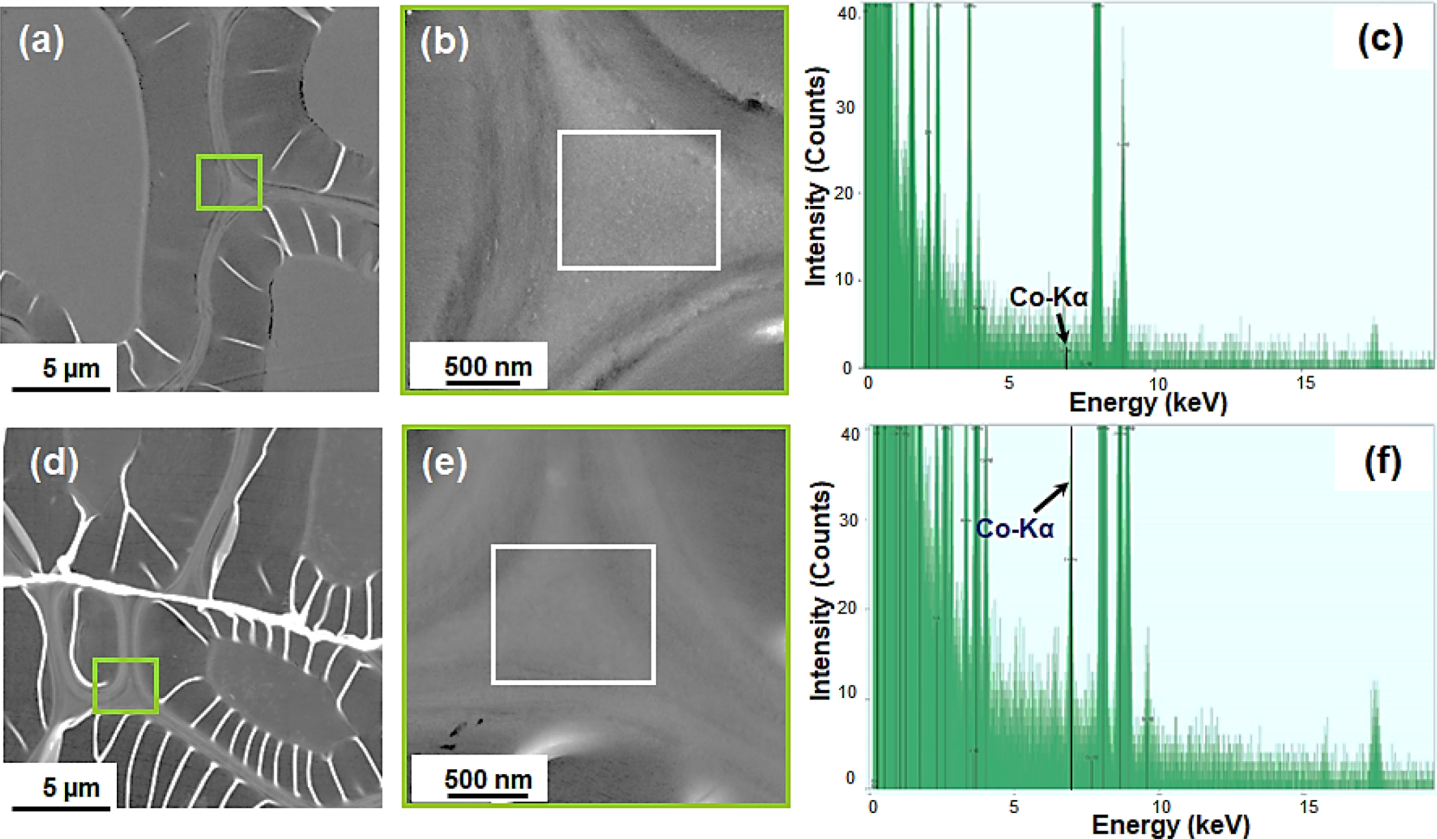
STEM-EDX analysis of the thin cross sections along the fiber direction of TW and TW0.1Co: (a) TW, (b) enlarged view near the cell wall corner of TW, (c) quantitative analysis of the presence of the Co element at the cell wall corner of TW, (d) TW0.1Co, (e) enlarged view near the cell wall of TW0.1Co, and (f) quantitative analysis of the presence of the Co element at the cell wall corner of TW0.1Co.

TW and C-TW0.1Co were also studied by using STEM-EDX (Figure 5). The kinks in both samples (bright lines) are artifacts from sample preparation. Figure 5a,b shows that the hierarchical morphology is preserved in the TW. The cell wall corner region is analyzed for cobalt signals. No nanoparticles were observed (Figure 5b), and a very weak Co signal (∼0 counts, Figure 5c) close to the noise level was recorded during the EDX mapping of this TW reference. In TW0.1Co (Figure 5d,e), any nanoparticles were not observed, but a strong Co signal (≥40 counts) was recorded during EDX mapping (Figure 5f). This is in support of no nanoparticle formation but, instead, cobalt ion coordination inside the wood structure.

### Mechanical and Optical Properties

**Figure 6:**
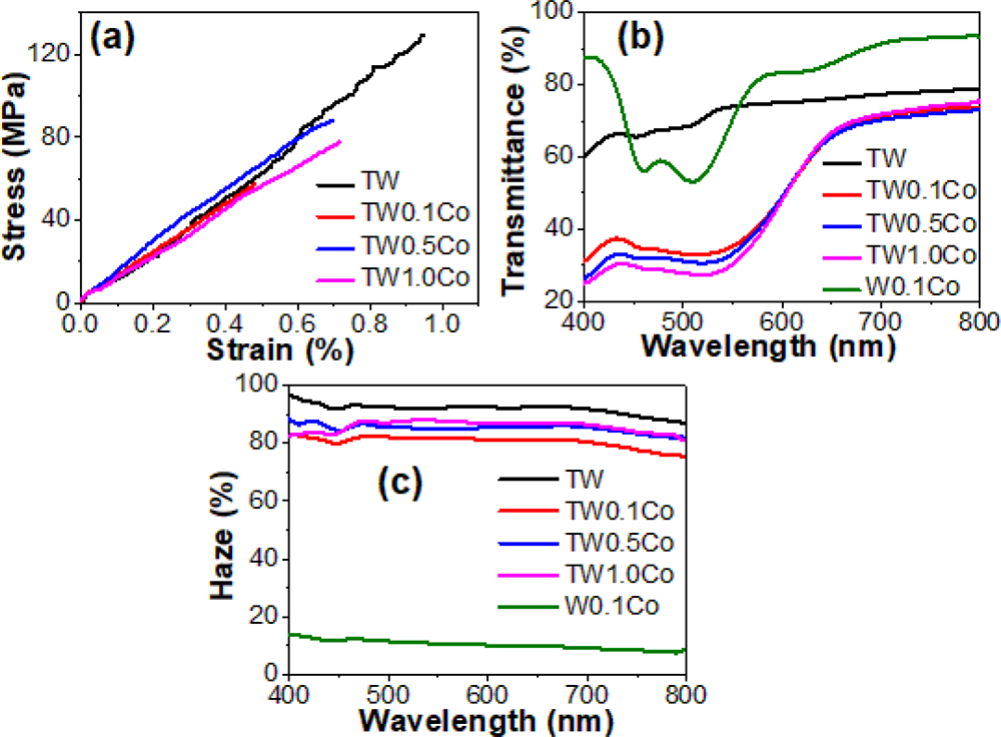
(a) Stress–strain curves for NW, BW, TW, and C-TWs, (b) optical transmittance, and (c) haze properties of TW, C-TWs, and 0.1 M cobalt salt solution (W0.1Co).

The stress and strain curves of NW, BW, TW, and C-TWs are shown in Figure 6a, with low strain to failure. BW shows a decreased tensile strength (41.3 ± 6.6 MPa) compared to that of NW (100.5 ± 11.3 MPa) (Figure S9) because of partial removal of lignin and hemicelluloses (Figure 2a). The increase in tensile strength was noticed when BW was impregnated by PMMA due to improved load transfer efficiency between cellulosic fibers and fibrils. The obtained tensile strength for TW was 119.4 ± 7.3 MPa (Figure 6a). This value is lower than in an earlier study on birch/PMMA,^37^ possibly due to the sensitivity of brittle composites to specimen preparation details. C-TWs showed lower tensile strength than TW. The tensile strengths of TW0.1Co, TW0.5Co, and TW1.0Co were 56.4 ± 4.2, 87.4 ± 3.4, and 77.7 ± 6.6 MPa, respectively. The NW showed an elastic modulus of 12.0 ± 2.3 GPa (Figure S9). The elastic moduli were similar in BW, TW, and C-TWs, taking data scatter into account.

The transmittance and haze of TW with a thickness of 1.1 mm and C-TWs with a thickness of 1.2 mm are shown in Figure 6b,c. TW with a wood weight fraction of 31% showed a transmittance of 74% and a haze of 92% at a wavelength of 550 nm (Table 2). This was slightly lower optical performance than thiol-ene-based^38^ or PLIMA-based^41^ systems due to larger RI mismatch between PMMA and wood cell walls and also from microdefects in the structure. Optical transmittance is the fraction of light traveling through the sample. Haze is a measure of transmitted light scattered at angles >2.5° from the incident beam. The cobalt ion composites TW0.1Co (wood wt, ∼30%), TW0.5Co (wood wt, ∼29%), and TW1.0Co (wood wt, ∼27%) showed distinct optical performance compared with TW (Figure 6b,c) due to the strong light absorption induced by cobalt ions. All the C-TWs showed a transmittance of ∼66% at a wavelength of 650 nm (Table 2) irrespective of cobalt ion content. The C-TWs showed slightly lower haze than TW. For TW0.1Co, TW0.5Co, and TW1.0Co, haze values were 82, 85, and 88%, respectively, at 550 nm (Table 2).

**Table 2:** Optical and Material Characteristics of C-TWs Based on Co Ion Introduction^a^

wood	chemical treatment	polymer	metal salt conc. (M)	thickness (mm)	*T* (%) at 550 nm	*T* (%) at 650 nm	*H* (%) at 550 nm	wood wt fraction (%)
birch	bleaching	PMMA	0.0	1.1	74	76	92	∼31
			0.1	1.2	36	67	82	∼30
			0.5	1.2	34	67	85	∼29
			1.0	1.2	31	66	88	∼27

a Note: *T* is for transmittance, and *H* is for haze.

**Figure 7:**
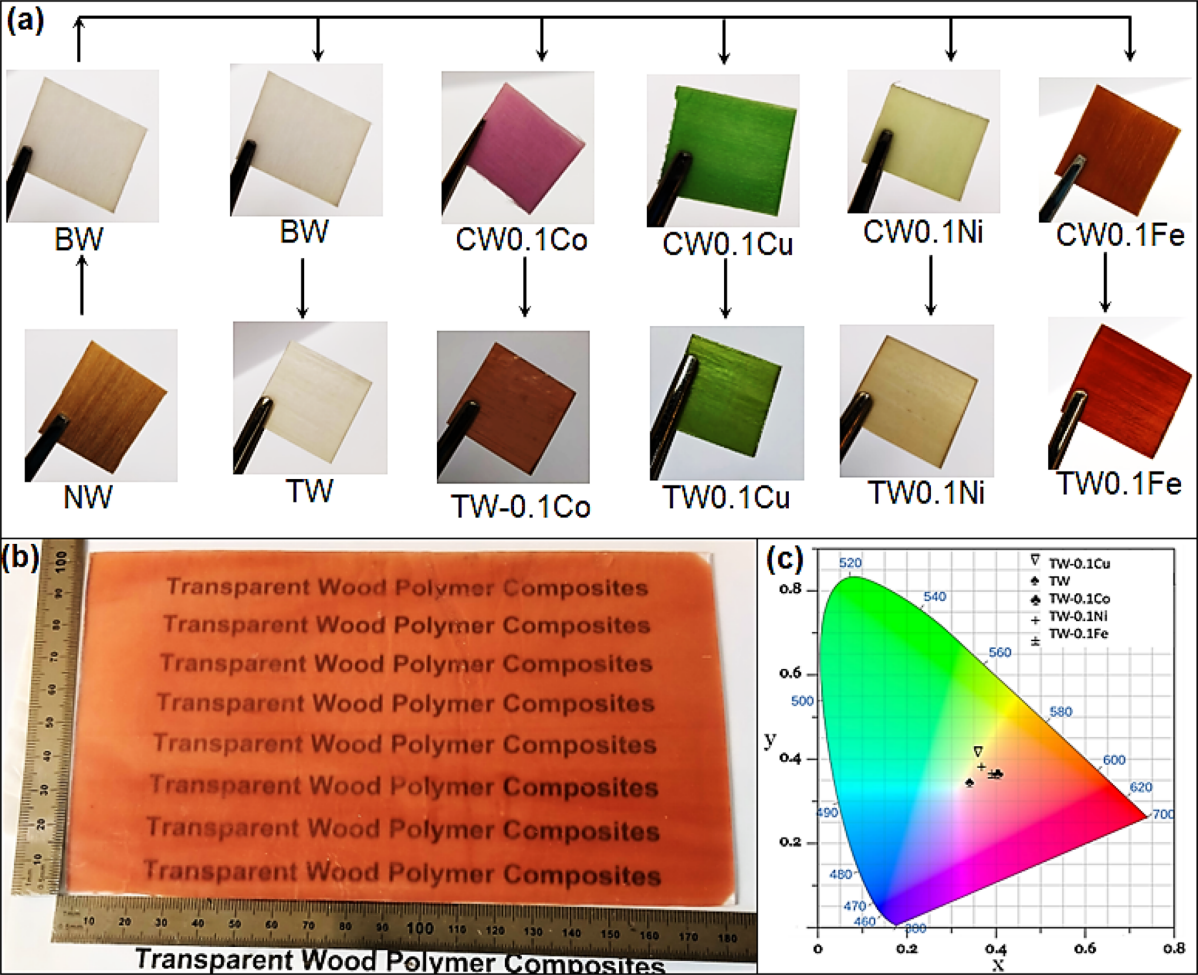
(a) Preparation of C-TWs from various metal salts, (b) scale-up of the colored transparent wood composite (TW0.1Co: 180 × 100 × 1 (*l* × *b* × *h*) mm^3^), and (c) CIE chromaticity chart of the C-TWs.

To investigate preparation of C-TWs from other metal salts, copper(II) chloride dihydrate, nickel(II) nitrate hexahydrate, and iron(III) chloride hexahydrate metal salts were used with a concentration of 0.1 M. The acetone-rinsed CWs prepared from copper(II) chloride dihydrate, iron(III) chloride hexahydrate, and nickel(II) nitrate hexahydrate metal salts appeared green, dark yellow, and light green, respectively (Figure 7a). The color of the wood template slightly changed after MMA infiltration and polymerization (Figure 7a). The optical properties of these C-TWs (thickness, 1.1 mm) are summarized in Figure S10 and Table S11. The C-TWs prepared from copper and nickel salts showed a transmittance of ∼58–60% and a haze of 83–85% at 550 nm. The C-TW prepared from iron salt showed slightly lower transmittance due to high light absorption in the visible range and 74% haze at 550 nm.

In practical applications, such as TW building materials, large-dimensional structures are required. To investigate feasibility aspects, colored transparent wood with a dimension of 180 × 100 × 1 (*l* × *b* × *h*) mm^3^ was fabricated with similar optical performance as small TW0.1Co samples (Figure 7b). The green hydrogen peroxide bleaching technology,^3^ a key step, is well established in the pulping industry, although conditions will be slightly different for veneer bleaching. To provide a more intuitive appreciation of the C-TWs color hues, the measured reflectance spectra (Figure S12) were converted to data points in the Commission Internationale de L’Eclairage (CIE) chromaticity chart (Figure 7c).

## Flame Retardancy

### Cone Calorimetry and Postcombustion Residue Analysis

**Figure 8:**
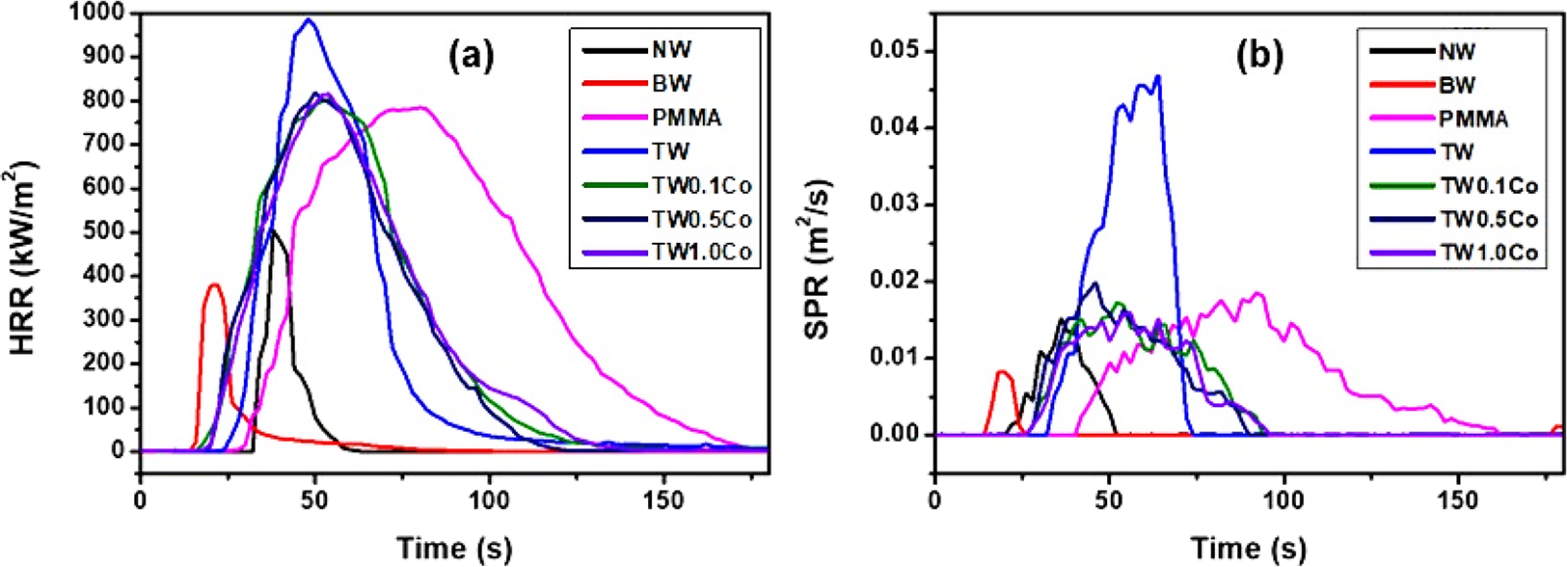
Heat release rate (HRR) vs time and (a) smoke production rate (SPR) vs time (b) of NW, BW, PMMA, TW, and C-TWs.

The flame-retardant characteristics of colored wood were investigated by cone calorimetry tests at 35 kW/m^2^ heat flux mimicking fires in early stages.^42^ Heat release rates and smoke production rates (HRR and SPR) vs time are evaluated and reported in Figure 8, while Table 3 collects average, peak, and integral parameters.

**Table 3:** Cone Calorimetry Test Results^a^

**sample**	**TTI** [s ± δ]	**HRR[kW/m**^**2**^**± δ]**	**pkHRR[kW/m**^**2**^**± δ]**	**THR[MJ/m**^**2**^**± δ]**	**EHC** [MJ/kg]	**TSR[m**^**2**^**/m**^**2**^**± δ]**	**residue** [% ± δ]
NW	32 ± 6	59 ± 18	523 ± 18	8.1 ± 2.5	13 ± 4	89 ± 15	∼0
BW	20 ± 1	76 ± 10	388 ± 38	5 ± 1	16.4 ± 3.2	25 ± 4	∼0
PMMA	35 ± 5	253 ± 3	796 ± 45	58 ± 5	24.6 ± 0.2	433 ± 75	∼0
TW	25 ± 1	189 ± 7	989 ± 73	33 ± 4	21 ± 1	444 ± 30	1 ± 1
TW0.1Co	19 ± 3	196 ± 14	850 ± 65	41 ± 4	23.0 ± 0.2	274 ± 50	5 ± 1
TW0.5Co	19 ± 1	199 ± 1	856 ± 67	38 ± 1	22.5 ± 0.5	270 ± 22	5 ± 1
TW1.0Co	19 ± 1	201 ± 2	827 ± 55	40 ± 3	21.1 ± 1.1	254 ± 18	5 ± 1

a TTI, time to ignition; HRR, heat release rate; pkHRR, peak HHR; THR, total heat released; TSR, total smoke released.

Early in the process, native birch starts to decompose, releasing highly flammable volatile gases, leading to sample ignition and flaming combustion. During this step, a charred carbonaceous residue is formed. After the flame is extinguished, this residue is then consumed by after-glowing phenomena (i.e., solid-state oxidation processes with emission of light). BW samples show a similar behavior with reduced pkHRR (peak heat release rate) and THR (total heat release) values due to the removal of lignin (Table 3). For PMMA-based transparent wood, the presence of the polymer increases the HRR and SPR as well as their related integral parameters. Indeed, neat PMMA burns vigorously, achieving high pkHRR values (≈800 kW/m^2^) while also releasing dense smoke, as demonstrated by TSR rate values (433 ± 75). Interestingly, the bleached wood substrate in TW controls ignition (occurs ≈10s earlier than for neat PMMA, Table 3).^43^ The wood substrate also changes the HRR plot shape and increases pkHRR compared with neat PMMA. PMMA is the main source for smoke production, and the contribution of the bleached wood substrate (BW) is almost negligible when comparing TSR values of PMMA, BW, and TW (Table 3). pkHRR values are slightly reduced by the presence of Co ions, indicating a limited effect on combustion rates, as also confirmed by EHC values that remain nearly unchanged.

**Figure 9:**
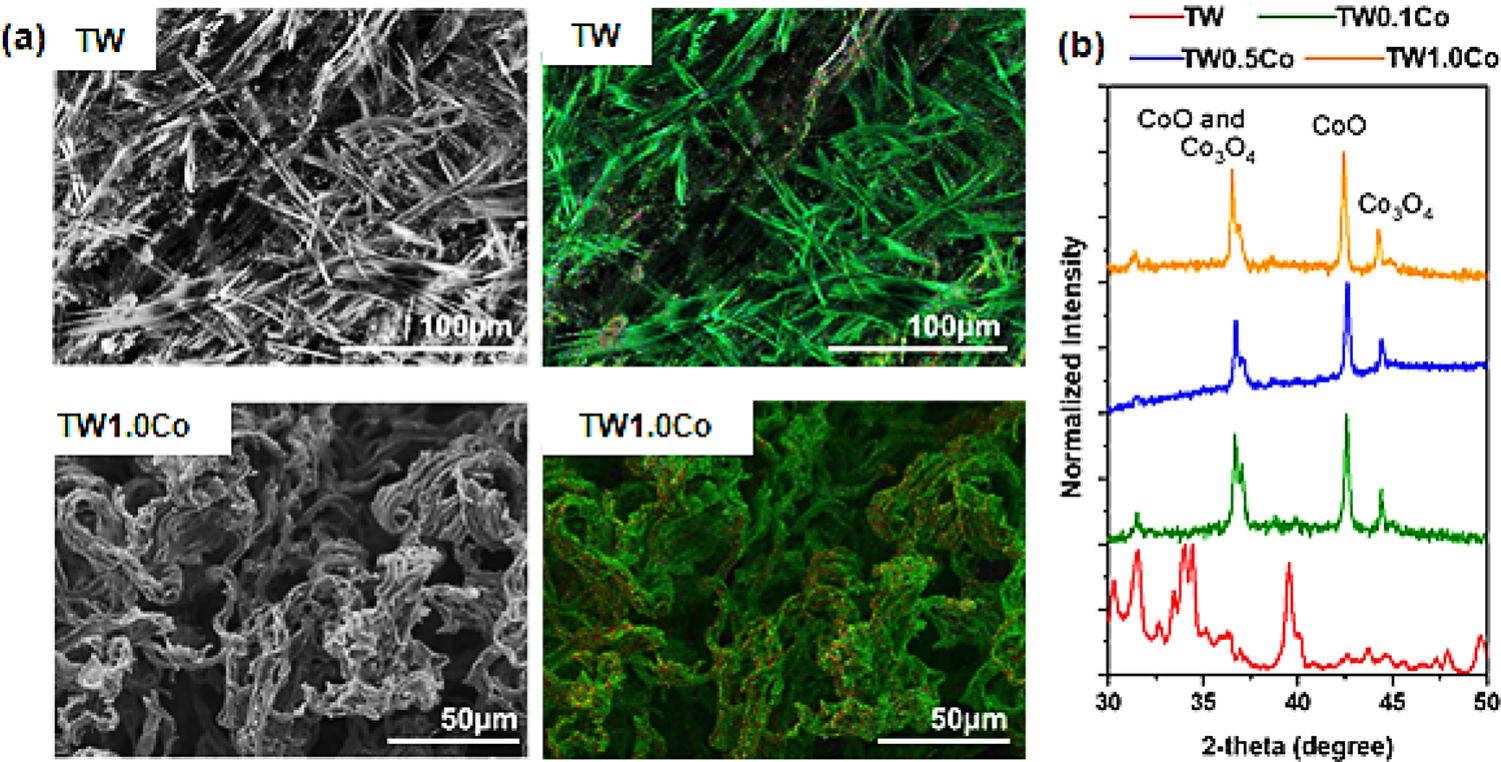
Compositional elemental analysis (EDS maps) of TW (C, O, and Na in red, green, and cyan, respectively) and TW1.0Co (Co, C, and O in yellow, blue, and magenta, respectively) of cone calorimetry test residue (a). XRD analysis of TW and C-TWs (b).

On the other hand, a beneficial effect is observed for SPR. Indeed, this latter is drastically lowered by 60% with a subsequent 40% decrease in TSR values (Table 3). These results can be ascribed to the presence of Co ions and their conversion, during combustion, to cobalt oxides,^44^ which have been reported to exert a catalytic effect on smoke reduction.^45^ The molar concentration of ions in the present range does not seem to affect the FR performances (see 0.1, 0.5, and 1 M sample data). There may be a threshold effect at 0.1 M or lower. Postcombustion residues were also investigated by means of SEM coupled with EDS microanalysis and XRD (Figure 9 and Figure S13).

SEM micrographs show inorganic needlelike structures encompassing metal cations (mostly Na) for TW samples. Conversely, wormlike char particles are observed for cobalt-containing samples. Co is homogeneously distributed within the structure. XRD plots support the formation of cobalt oxides (CoO and Co_3_O_4_).^46^ This confirms the starting hypothesis where the catalytic effects of cobalt oxides toward smoke were deemed responsible for the measured reduction in smoke production. In addition, the observed charred morphology can be ascribed to an improved char formation from the cellulose template due to Co ions.^47^ This phenomenon, along with the production of a ceramic cobalt oxide barrier, can be correlated with the observed slight reduction in HRR values. SEM and XRD results thus confirm that the production of cobalt oxides that have been hypothesized as the reduced smoke production rates and total smoke release from metal ions is of great importance in terms of fire safety. Statistics on fire accidents point out smoke as one of the main casualty reason due to incapacitating action on people trying to escape, as well as long-terms health effects from smoke exposure.^48−50^

## Conclusions

Bleached wood was impregnated by metal ions and then successfully used to prepare C-TWs with good mechanical load-bearing properties. These C-TWs with metal ions combined diffuse light transmission with aesthetic coloration and a strongly reduced smoke production rate. Cobalt ions were sorbed inside the cell wall. They were preferably distributed in lignin-rich areas such as the middle lamellae and cell wall corners. Most likely, they have ionic interaction with electronegatively charged wood polymers since they remain after washing. The depth of color was adjustable by changing the metal salt concentration and, thus, the weight fraction of ions in the material. The color is readily tailorable by changing the type of metal salt, and the fabrication concept has scaling potential.

The hierarchical structure of the wood reinforcement is of critical importance. Large tubular pores (20–200 μm diameter in birch) facilitate liquid flow during impregnation. In addition, the nanoscale cell wall porosity allows for the sorption of functional molecules. This hierarchically structured substrate from renewable resources provides opportunities for a large variety of modifications for interesting optical effects in cellulosic biocomposites.

## Supporting Information Available

Figure S1: prepared metal salts and DI water solution; Figure S2: cross-sectional micrographs of native wood (NW) and bleached wood (BW); Figure S3: NW, BW, and colored wood (CW); Figure S4: cross-sectional view of C-TWs; Figure S5: SEM with EDX analysis of the cross section of TW; Figure S6: C-TWCs under SEM with relative mapping of the “Co” element in EDX analysis; Figure S7: cobalt ions contained in weight percentage in CW; Figure S8: wide-angle X-ray diffraction patterns of TW and C-TWs; Figure S9: stress–strain curves for NW and BW; Figure S10: optical properties of C-TWs prepared from other metal salts excluding cobalt(II) nitrate hexahydrate; Table S11: optical properties and material characteristic of C-TWs prepared from other metal salts excluding cobalt(II) nitrate hexahydrate; Figure S12: reflectance of the C-TWs; Figure S13: compositional elemental analysis (EDS maps) of C-TWs of cone calorimetry test residue (PDF)
