# Supplementary material for: Coloration and Fire Retardancy of Transparent Wood Composites by Metal Ions
Source: ACS Appl Mater Interfaces. 2023 Dec 6;15(50):58850–60. doi: 10.1021/acsami.3c13585 (PMC10739590; doi:10.1021/acsami.3c13585)
Supplement: Supplementary file 1 — am3c13585_si_001.pdf [file am3c13585_si_001.pdf]

Supporting information

Coloration and Fire Retardancy of Transparent Wood Composites by Metal Ions

Pratick Samanta [1*], Archana Samanta [2], Lorenza Maddalena [3], Federico Carosio [3], Ying Gao[1,4], Céline Montanari[1], Mathias Nero[5], Tom Willhammar[5], Lars A. Berglund[1*] and Yuanyuan Li[1*]

[1]Department of Fibre and Polymer Technology, Wallenberg Wood Science Center, KTH Royal Institute of Technology, Stockholm 100 44, Sweden

[2]Department of Applied Physics, KTH Royal Institute of Technology, Stockholm 114 19, Sweden

[3]Dipartimento di Scienza Applicata e Tecnologia, Politecnico di Torino, Alessandria Campus, Viale Teresa Michel 5, 15121 Alessandria, Italy

[4]Jiangsu Co-Innovation Center of Efficient Processing and Utilization of Forest Resources, Nanjing Forestry University, Nanjing 210037, China

[5]Department of Materials and Environmental, Chemistry, Stockholm University, SE-106 91 Stockholm, Sweden

*E-mail: praticksamanta@gmail.com, yua@kth.se and blund@kth.se

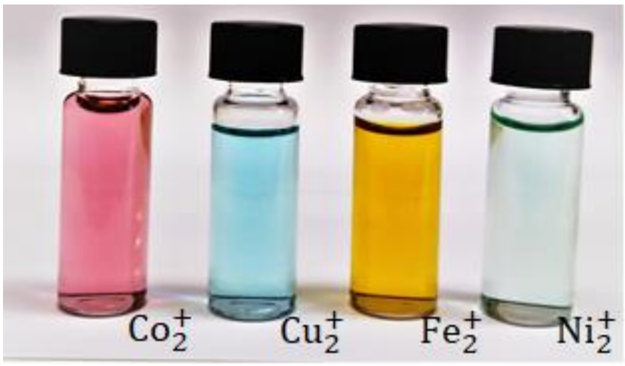

Figure S1: 0.1 M solutions were prepared with different metal slats (cobalt (II) nitrate hexahydrate, copper (II) chloride dihydrate, iron (III) chloride hexahydrate and nickel (II) nitrate hexahydrate) and DI-water.

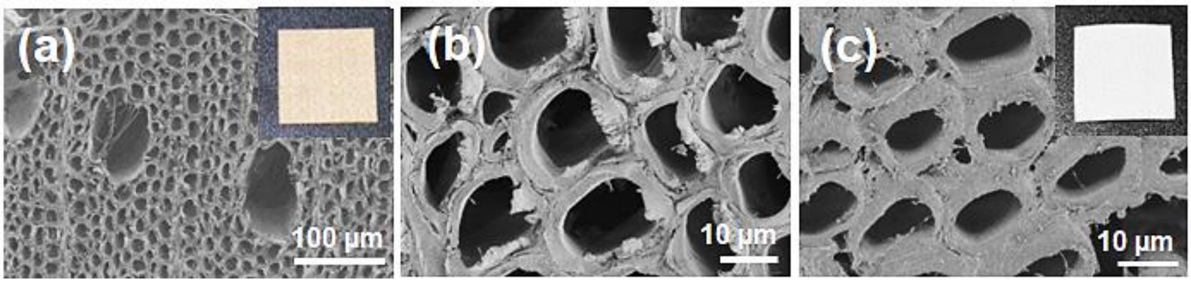

Figure S2: Cross-section micrographs of native wood (NW) and bleached wood (BW) (a) NW, (b) enlarged view of NW cross-section, (c) BW.

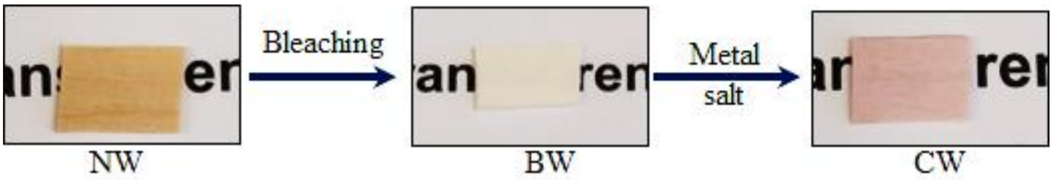

Figure S3: The native wood (NW), bleached wood (BW) and colored wood (CW) prepared from cobalt salt are placed on printed paper.

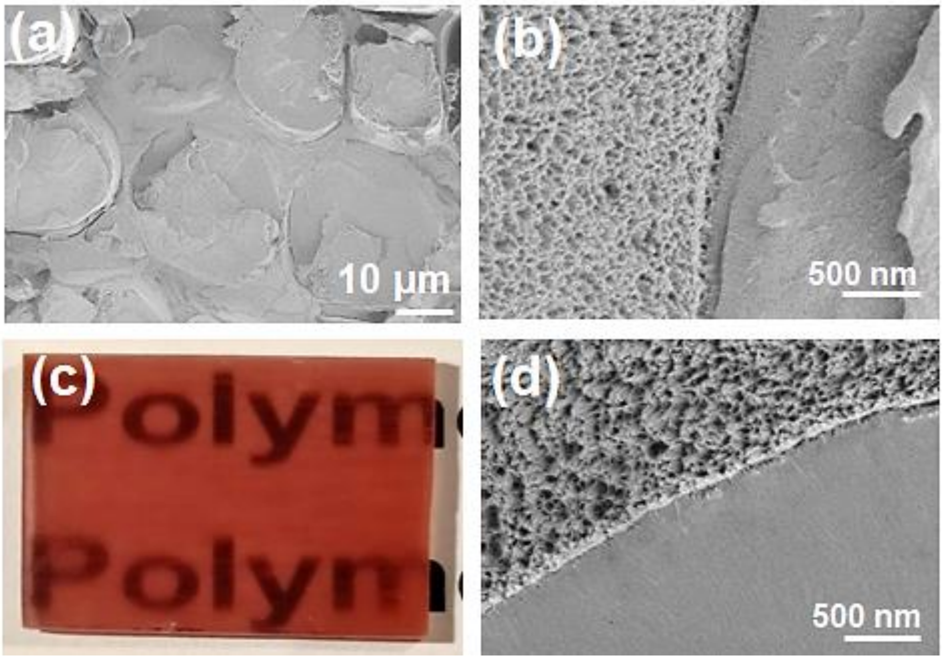

Figure S4: The cross-section view of (a) TW0.5Co, (b) enlarged view of TW0.5Co cell wall. Colored transparent wood placed on printed paper (c) TW0.5Co and (d) enlarged view of cross-section of TW1.0Co cell wall.

‘

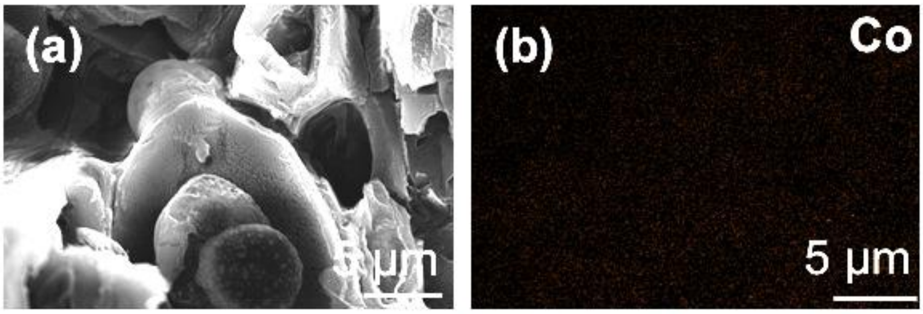

Figure S5: SEM with EDX analysis of the cross-section of TW specimen (a) surface of cross-section of TW under SEM (b) surface mapped with ‘Co’ element in EDX analysis.

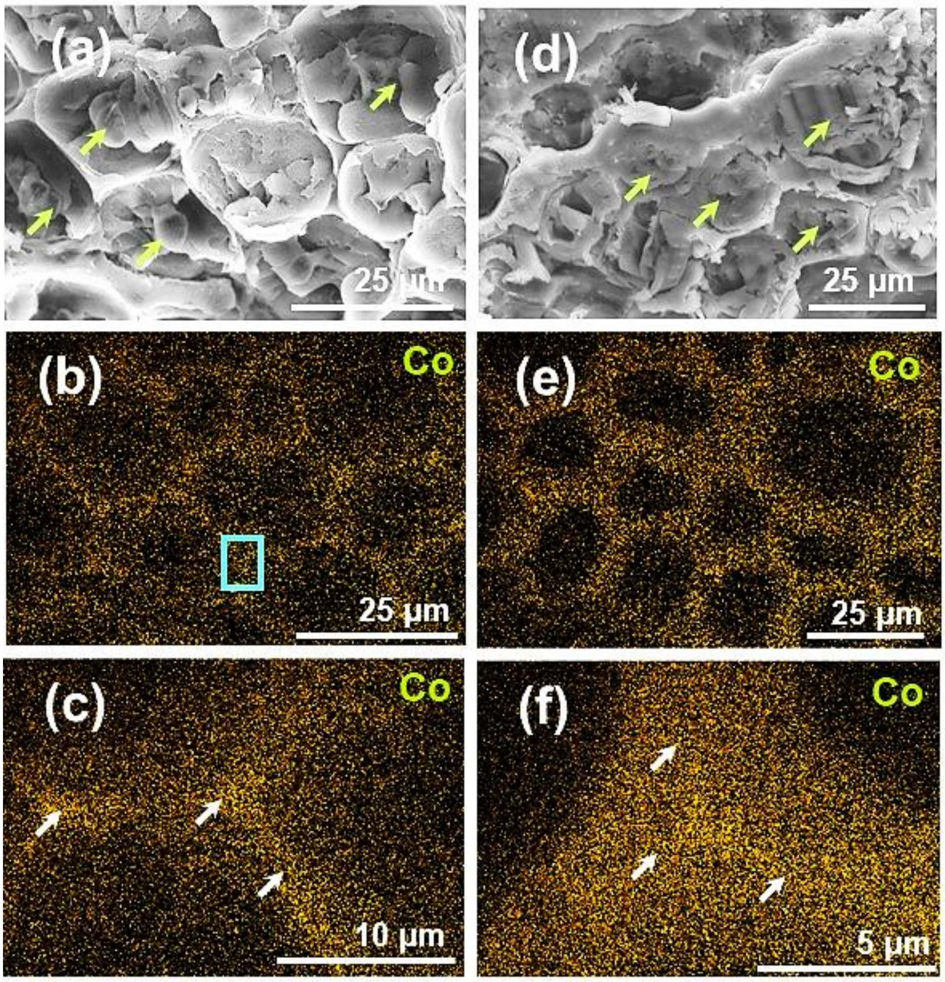

Figure S6: Colored transparent wood composites (C-TWs) under SEM with relative mapping of Co element in EDX analysis. (a) TW0.5Co (b) relative mapping of Co element of TW0.5Co, (c) enlarged view of TW0.5Co middle lamella region, (d) TW1.0Co (e) relative mapping of Co element of TW1.0Co and (f) enlarged view of TW1.0Co cell wall corner region.

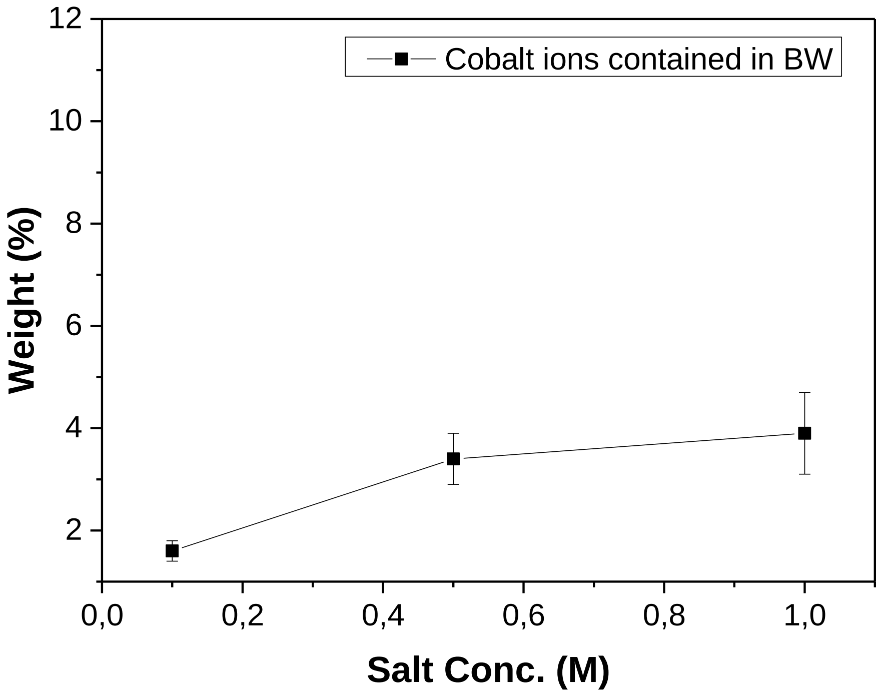

Figure S7: The cobalt ions contained in weight percentage in CW with respect to salt concentration.

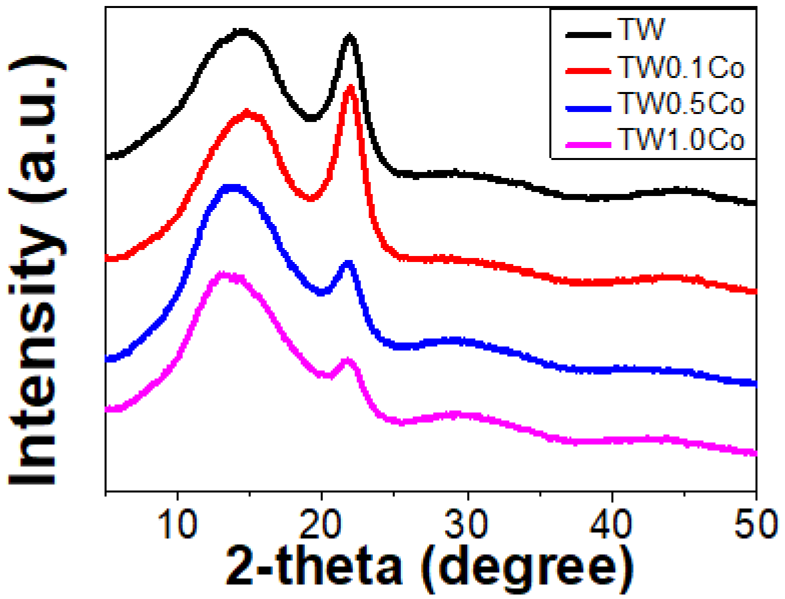

Figure S8: Wide angle x-ray diffraction patterns of TW and C-TWs

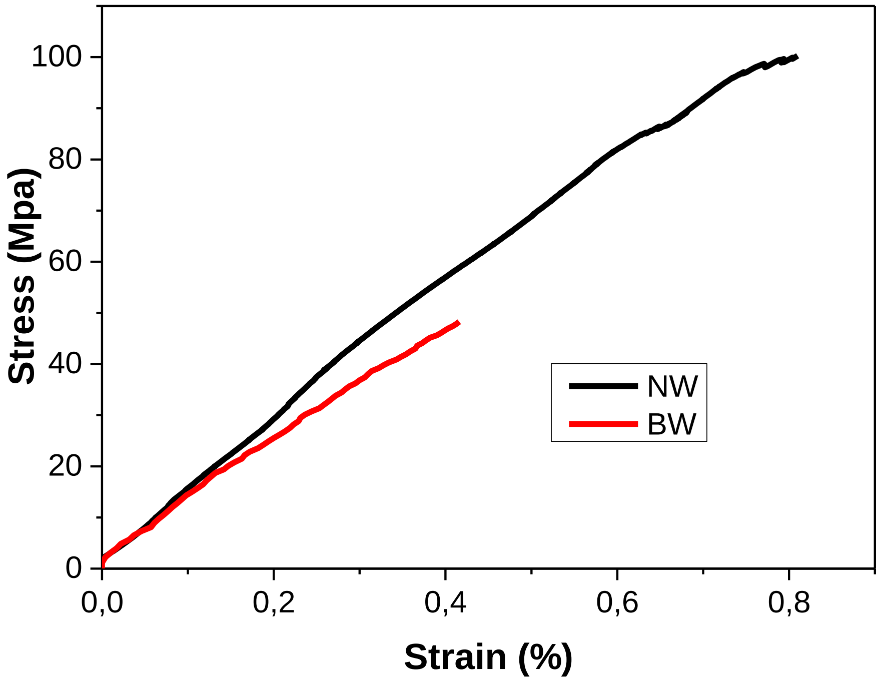

Figure S9: (a) stress-strain curves for NW and BW.

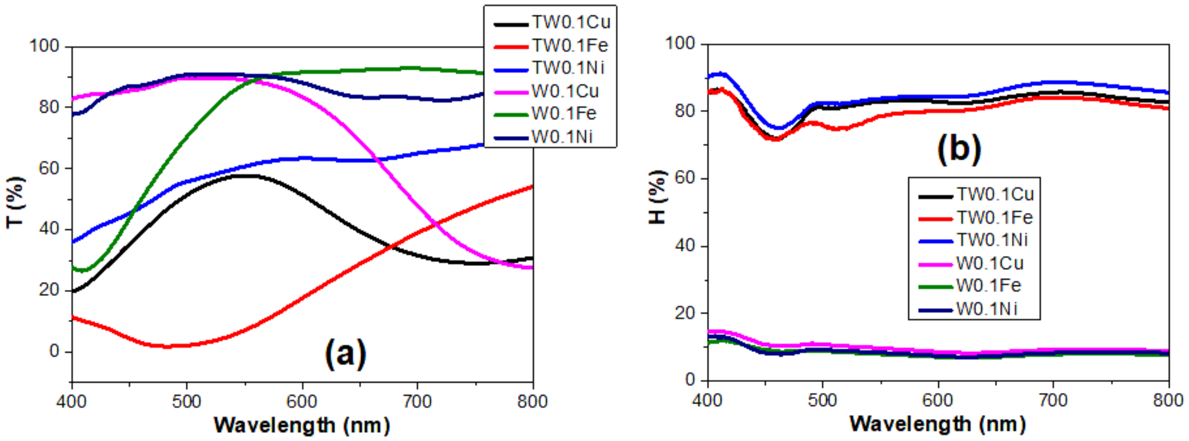

Figure S10: The optical properties of C-TWs prepared copper (II) chloride dihydrate, nickel (II) nitrate hexahydrate and iron (III) chloride hexahydrate salt solutions. 0.1 M solutions each salt was used to prepare C-TWs. (a) optical transmittance and (b) haze behavior

Table S11: Optical properties and material characteristic of colored transparent wood.

Wood	Chemical treatment	polymer	Metal salt conc. (0.1M)	Thickness (mm)	T (%) at 550 nm	T (%) at 650 nm	H (%) at 550 nm
Birch	Bleaching	PMMA	Copper(II) chloride dehydrate	1.1	58	38	83
			Iron(III) chloride hexahydrate	1.1	7	34	74
			Nickel(II) nitrate hexahydrate	1.1	60	62	85

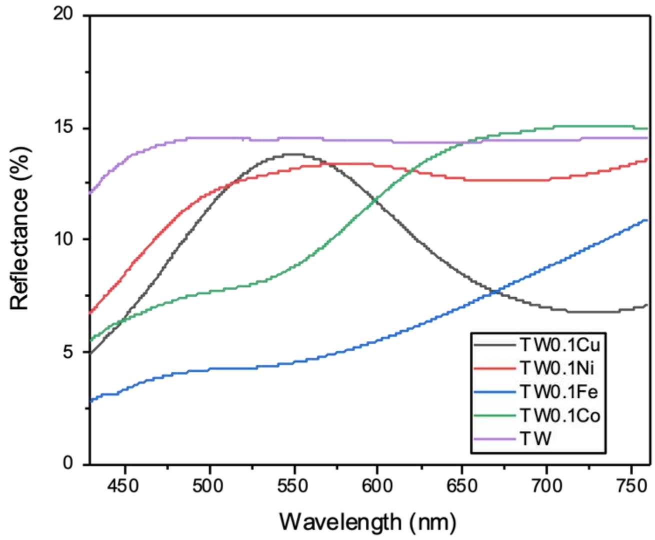

Figure S12: The reflectance of C-TWs

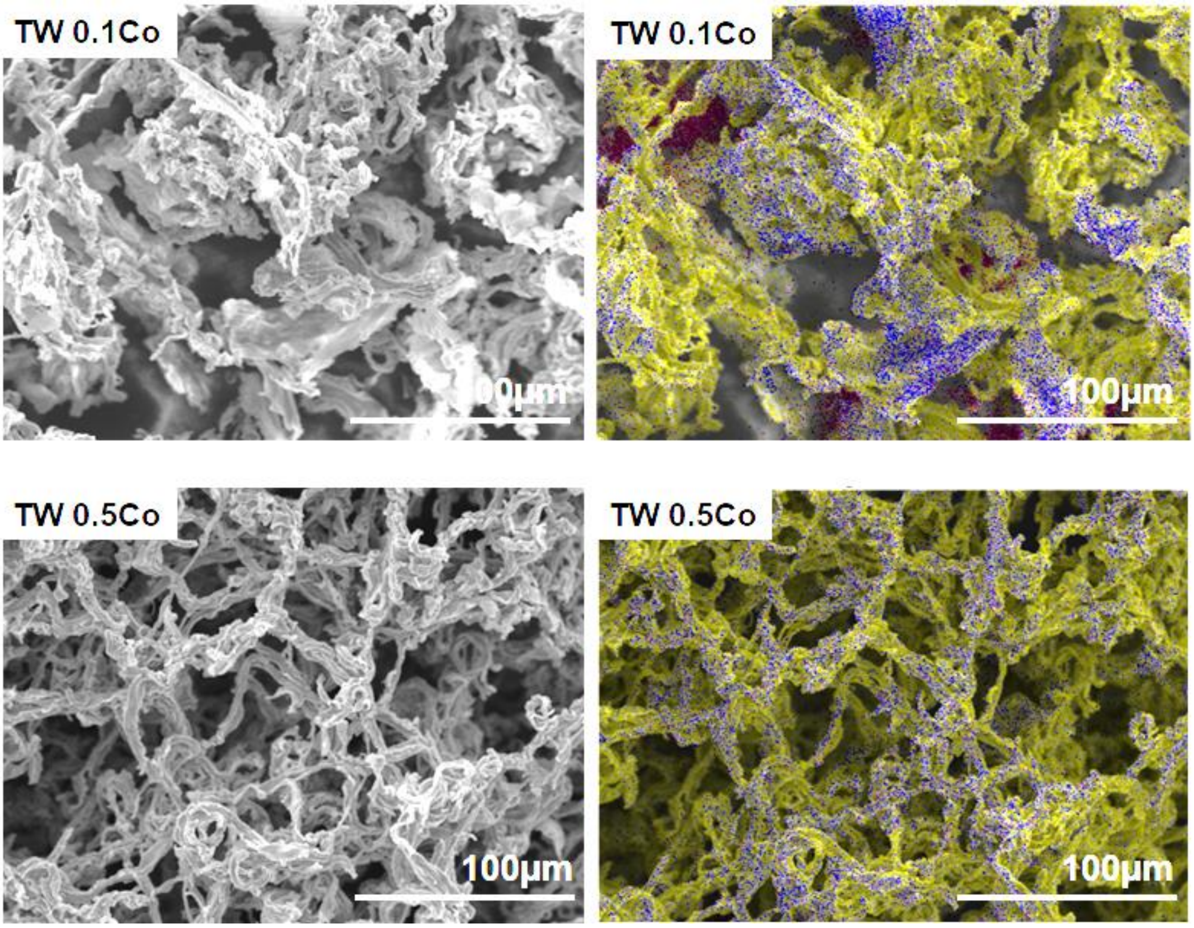

Figure S13: Compositional elemental analysis (EDS maps) of TW0.1Co and TW0.5Co (Co, C, O in yellow, blue and magenta, respectively) of cone calorimetry test residue.
